# Draft Genome Sequence of *Campylobacter corcagiensis* Strain CIT045^T^, a Representative of a Novel *Campylobacter* Species Isolated from Lion-Tailed Macaques (*Macaca silenus*)

**DOI:** 10.1128/genomeA.00248-14

**Published:** 2014-04-17

**Authors:** Monika Koziel, Alan Lucid, Susan Bullman, Gerard D. Corcoran, Brigid Lucey, Roy D. Sleator

**Affiliations:** a Department of Biological Sciences, Cork Institute of Technology, Bishopstown, Cork, Ireland; b Department of Microbiology, Cork University Hospital, Wilton, Cork, Ireland

## Abstract

*Campylobacter corcagiensis* CIT045^T^ (=CCUG 64942^T^, LMG 27932^T^), a new member of the *Campylobacter* genus, has recently been isolated from lion-tailed macaques in Cork, Ireland. To further characterize this new species and its potential pathogenicity, the genome sequence of *C. corcagiensis* was determined and is presented here.

## GENOME ANNOUNCEMENT

The *Campylobacter* genus currently includes 25 species and 8 subspecies that have been isolated from humans, birds, and domestic animals (1). Many of the *Campylobacter* spp. described to date have been shown to cause disease in both humans and animals (1). *Campylobacter corcagiensis* was isolated by our research group from captive primates. The taxonomic position of this species is currently being described; however, its virulence and pathogenicity are as yet unknown. Preliminary studies in our lab have shown that *C. corcagiensis* possesses some interesting phenotypic characteristics, such as bile and salt tolerance, as well as resistance to certain antibiotics, such as metronidazole and nalidixic acid (unpublished data). To further elucidate its pathogenic potential, whole-genome sequencing of this strain was performed.

*C. corcagiensis* strain CIT045^T^ (=CCUG 64942^T^, LMG 27932^T^) was sequenced using Illumina MiSeq with 250-bp paired-end reads. The sequencing generated 794,990 reads in pairs and an estimated genome coverage of approximately 200×. The reads were assembled *de novo* using the Velvet (version 1.2.10) assembly tool (2) into 21 contigs. The total draft genome size is 1,676,909 bp, and the estimated G+C content is 31.9 mol%. The genome was annotated using the automated annotation server RAST (3). A total of 1,748 coding sequences (CDS) were identified, with 578 assigned as hypothetical, accounting for 33% of all CDS. From those, a total of 125 proteins were predicted to be secreted using SignalP 4.1 (4).

Among the predicted CDS, several putative virulence factors were identified, including genes encoding putative efflux pumps involved in conferring antibiotic resistance, such as the *cmeABC* operon and the macrolide-specific efflux operon *macAB* (5, 6). Moreover, putative genetic loci that have been associated with resistance to quaternary ammonium compounds (*sugE*) have been found in the genome (7).

The genome of *C. corcagiensis* also contains other putative virulence genes associated with the formation of type IV pili (*pilT*, *pilQ*) (8), adhesion (fibronectin-fibrinogen binding protein gene) (9), invasion (*ciaB*) (10), or increased intestinal permeability (zonula occludens toxin gene) (11). These are potentially involved in promoting gastrointestinal pathogenicity; however, further studies are required to investigate the true virulence and pathogenic potential of this strain.

### Nucleotide sequence accession numbers.

This whole-genome shotgun project has been deposited at DDBJ/EMBL/GenBank under the accession no. JFAP00000000. The version described in this paper is version JFAP01000000.
